# PEDF inhibits lymphatic metastasis of nasopharyngeal carcinoma as a new lymphangiogenesis inhibitor

**DOI:** 10.1038/s41419-021-03583-1

**Published:** 2021-03-17

**Authors:** Chuanghua Luo, Haofan Yin, Tianxiao Gao, Caiqi Ma, Junxi Liu, Ting Zhang, Zumin Xu, Xi Wang, Danrui Zhang, Weiwei Qi, Zhonghan Yang, Guoquan Gao, Xia Yang, Ti Zhou

**Affiliations:** 1 Program of Molecular Medicine, Affiliated Guangzhou Women and Children’s Hospital, Zhongshan School of Medicine, Sun Yat-sen University, Guangzhou, China; 2 Department of Biochemistry, Zhongshan School of Medicine, Sun Yat-sen University, Guangzhou, China; 3 State Key Laboratory of Oncology in South China, Collaborative Innovation Center for Cancer Medicine, Sun Yat-sen University Cancer Center, Guangzhou, China; 4 Department of Laboratory Medicine, Guangzhou First People’s Hospital, School of Medicine, South China University of Technology, Guangzhou, China; 5 Cancer Center, Affiliated Hospital of Guangdong Medical College, Zhanjiang, China; 6 Guangdong Engineering & Technology Research Center for Gene Manipulation and Biomacromolecular Products, Sun Yat-sen University, Guangzhou, China; 7 Guangdong Province Key Laboratory of Brain Function and Disease, Zhongshan School of Medicine, Sun Yat-sen University, Guangzhou, China; 8 China Key Laboratory of Tropical Disease Control (Sun Yat-sen University), Ministry of Education, Guangzhou, China

**Keywords:** Metastasis, Head and neck cancer

## Abstract

Nasopharyngeal carcinoma (NPC) is one of the most malignant tumors in southern China and Asia, and lymph node metastasis is an important cause for treatment failure. Lymphangiogenesis is a crucial step in lymphatic metastasis of NPC, while little is known about lymphangiogenesis in NPC. Similar to angiogenesis, lymphangitic neovascularization is a process of balance between pro-lymphangiogenesis and anti-lymphangiogenesis factors, but there are few studies on endogenous lymphangiogenesis inhibitors. Pigment epithelium-derived factor (PEDF) is a well-known effective endogenous angiogenesis inhibitor. However, the relationship between PEDF and lymphangiogenesis remains unknown. Our present study reveals that PEDF is lowly expressed in human NPC tissues with poor prognosis and is negatively correlated with lymphatic vessel density (LVD). Consistently, PEDF inhibits lymphangiogenesis and lymphatic metastasis of NPC in vivo experiments. Mechanistically, PEDF inhibits the proliferation, migration, and tube formation of lymphatic endothelial cells and promotes cell apoptosis. On the other hand, PEDF reduces the expression and secretion of vascular endothelial growth factor C (VEGF-C) of NPC cells through the nuclear factor-κB (NF-κB) signaling pathway. Our findings indicate that PEDF plays a vital role in lymphatic metastasis by targeting both lymphatic endothelial cells and NPC cells, and PEDF may represent a novel therapeutic target for NPC.

## Introduction

Nasopharyngeal carcinoma (NPC) is an upper respiratory tract tumor arising from nasopharynx epithelium, which is common in southern China and Asia. It is estimated that 129,079 new cases and 72,987 deaths are due to NPC in the world in 2018, accounting for 0.7% of new cancer cases and 0.8% of all cancer-related deaths, respectively, while China is accounting for 47.7% of the new NPC cases in the world^1,2^. Compared with other head and neck cancers, NPC is prone to have lymphatic metastasis because of its well-developed network of lymphatics^3^. In all, 70–80% of NPC patients have developed cervical lymph node metastases when they are first diagnosed, and the high incidence of early lymph node metastasis and distant metastasis resulted in 15 to 42% failure of treatment^4–7^. However, it is a great pity that a proper solution to lymph node metastasis of NPC is rare.

Lymphatic metastasis is usually accompanied by lymphangiogenesis and lymphatic remodeling. Lymphangiogenesis is the outgrowth of new lymphatics from the preexisting lymphatic vessels, which gains wide attention recently for its involvement in lymphatic metastasis^8–10^. Lymphangiogenesis is mainly induced by vascular endothelial growth factor C (VEGF-C), VEGF-D, and their receptor VEGFR3, and their interactions will lead to the proliferation and migration of lymphatic endothelial cells and, finally, the formation of lymphatic vessels^11–13^. Except for VEGF-C/D, many factors were reported to promote tumor lymphangiogenesis, such as VEGF-A^14,15^, hepatocyte growth factor (HGF)^16^, fibroblast growth factor(FGF)^17^, epidermal growth factor(EGF)^18^, platelet-derived growth factor-A(PDGF-A)^19^, and insulin-like growth factor(IGF)^20^. Just like angiogenesis, there is a balance between lymphangiogenic inducers and inhibitors to maintain a largely quiescent lymphatic vasculature under physiologic conditions^8,21^. But there are few studies on endogenous lymphangiogenesis inhibitors, and the reported endogenous lymphangiogenesis inhibitors include collagen fragments such as endostatin^22,23^, thrombospondin-1 (TSP-1)^24,25^, sVEGFR-2s^26^, kallistatin^27,28^, and MMP^29^. It is necessary to find a better and more effective endogenous lymphangiogenesis inhibitor, and the role and mechanism of these inhibitors still need to be further elucidated.

Pigment epithelium-derived factor (PEDF) is a serine proteinase inhibitor, a member of the serpin superfamily, which is recognized as a potent inhibitor of angiogenesis^30,31^. After several decades of research, more and more biological functions of PEDF have been revealed. Studies have shown that PEDF has neuroprotection, promoting neural stem cells to self-renew, anti-inflammation, regulating lipid metabolism, and other biological functions^32–34^. However, whether PEDF participates in the inhibition of lymphangiogenesis remains unknown. There are many similar processes between angiogenesis and lymphangiogenesis. Therefore, we speculate that PEDF may inhibit lymphangiogenesis like other angiogenesis inhibitors, endostatin, and TSP-1.

Based on these premises, in this study, we investigated whether PEDF inhibited lymphangiogenesis and lymphatic metastasis in NPC and elucidated the possible mechanism.

## Results

### Low expression of PEDF is correlated with poor prognosis and negatively with LVD in human NPC tissues

**Fig. 1:**
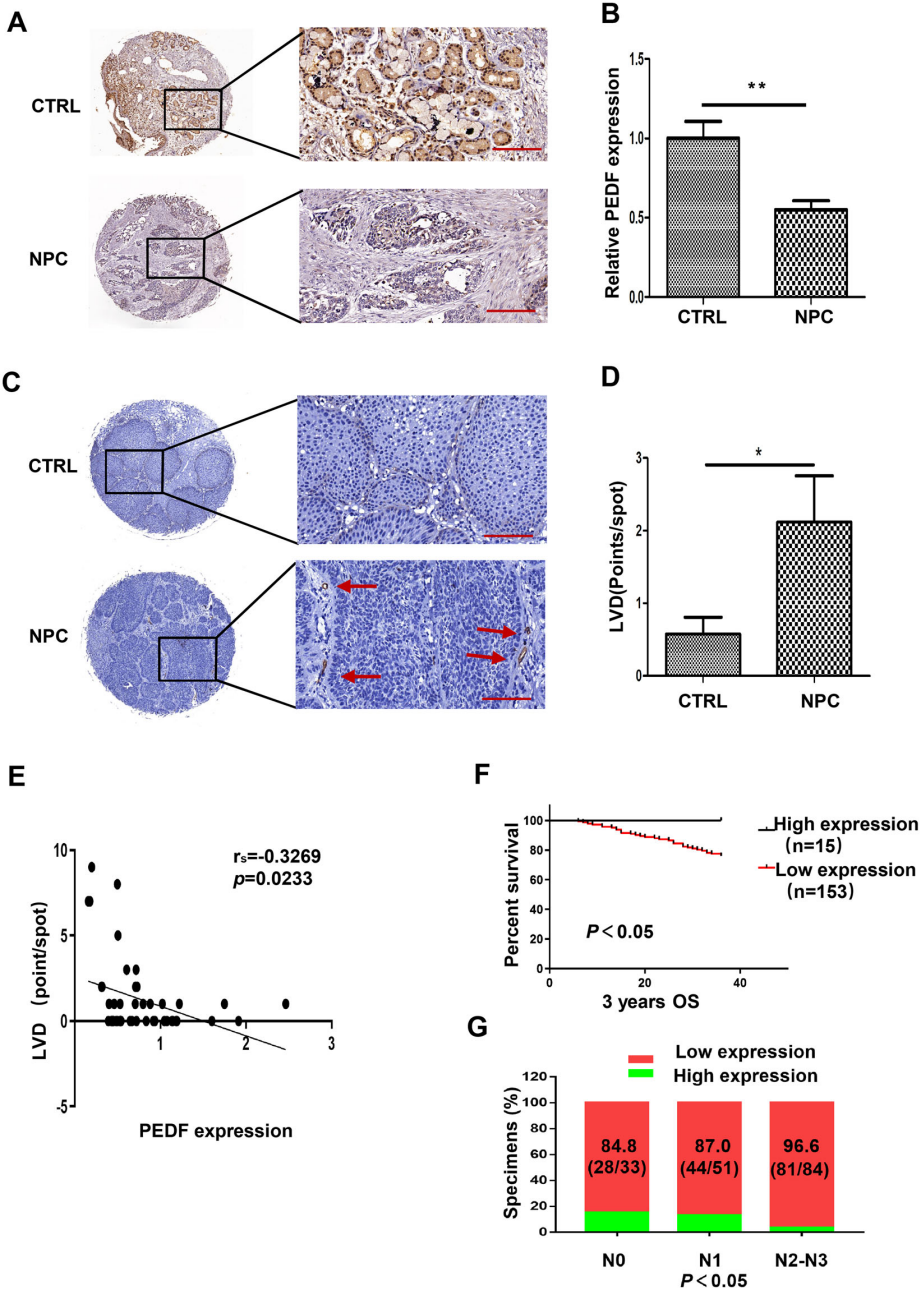
Low expression of PEDF is correlated with poor prognosis and negatively with LVD in human NPC tissues. **A** PEDF staining of tissue microarray by IHC (image magnification, 200×), the tissue microarray contains human nasopharyngeal cancer (22 cases), papilloma (15 cases), polyp (6 cases), hyperplasia, and inflammation (6 cases). The malignant tumor group includes human nasopharyngeal cancer (22 cases). The others (27 cases) are the control group. **B** The histogram represents the PEDF expression in the tissue microarray quantified by grayscale scanning with Image pro-plus. **C** LVD in the tissue microarray with D2-40 staining lymphatic tubes (image magnification, 200×) and the statistical graph (**D**). **E** The correlation analyses of PEDF expression and LVD in the tissue microarray. **F** Kaplan–Meier analysis of 3-years overall survival (OS) and **G** proportion of patients at different lymph node stages in a set of 168 nasopharyngeal cancer patients (*n* = 168) according to PEDF expression. Scale bars:100 μm (**A**, **C**), *p < 0.05, **p < 0.01, the results are presented as mean ± SD.

To find the relationship between PEDF and LVD in NPC, we detected the PEDF expression and LVD by immunohistochemistry (IHC) in a NPC tissue microarray, which contains human nasopharyngeal cancer (22 cases), papilloma (15 cases), polyp (6 cases), hyperplasia, and inflammation (6 cases). The result revealed that the PEDF expression in NPC tissues was lower than the control tissues (Fig. 1A, B). On the contrary, LVD in NPC tissues was higher than in the control tissues (Fig. 1C, D). Furthermore, the Spearman rank correlation analysis revealed that PEDF was negatively correlated with LVD in human NPC tissues, and the Spearman *r* value was −0.3269 (Fig. 1E).

Next, we further collected 168 NPC tissue samples with sufficient clinic information and detected PEDF expression with IHC, and then these tumors were then categorized as positive and negative PEDF groups. We found that NPC patients with negative PEDF expression had advanced pathological tumors and clinical stages (Supplementary Table 1). The Kaplan–Meier analysis and log-rank test showed that patients with positive PEDF expression were better than negative PEDF patients in 3-years survival (Fig. 1F). In addition, the percentage of PEDF with low expression was increased gradually from N0 (84.8%) to the N2–3 stage (96.6%) (Fig. 1G), suggesting that PEDF was negatively correlated with the lymph node stage. These findings indicated that PEDF was negatively correlated with LVD, and downregulation of PEDF was correlated with poor prognosis in human NPC tissues.

### Recombinant PEDF reduces the LVD and lymph node metastasis in the cell-derived xenograft animal model

**Fig. 2:**
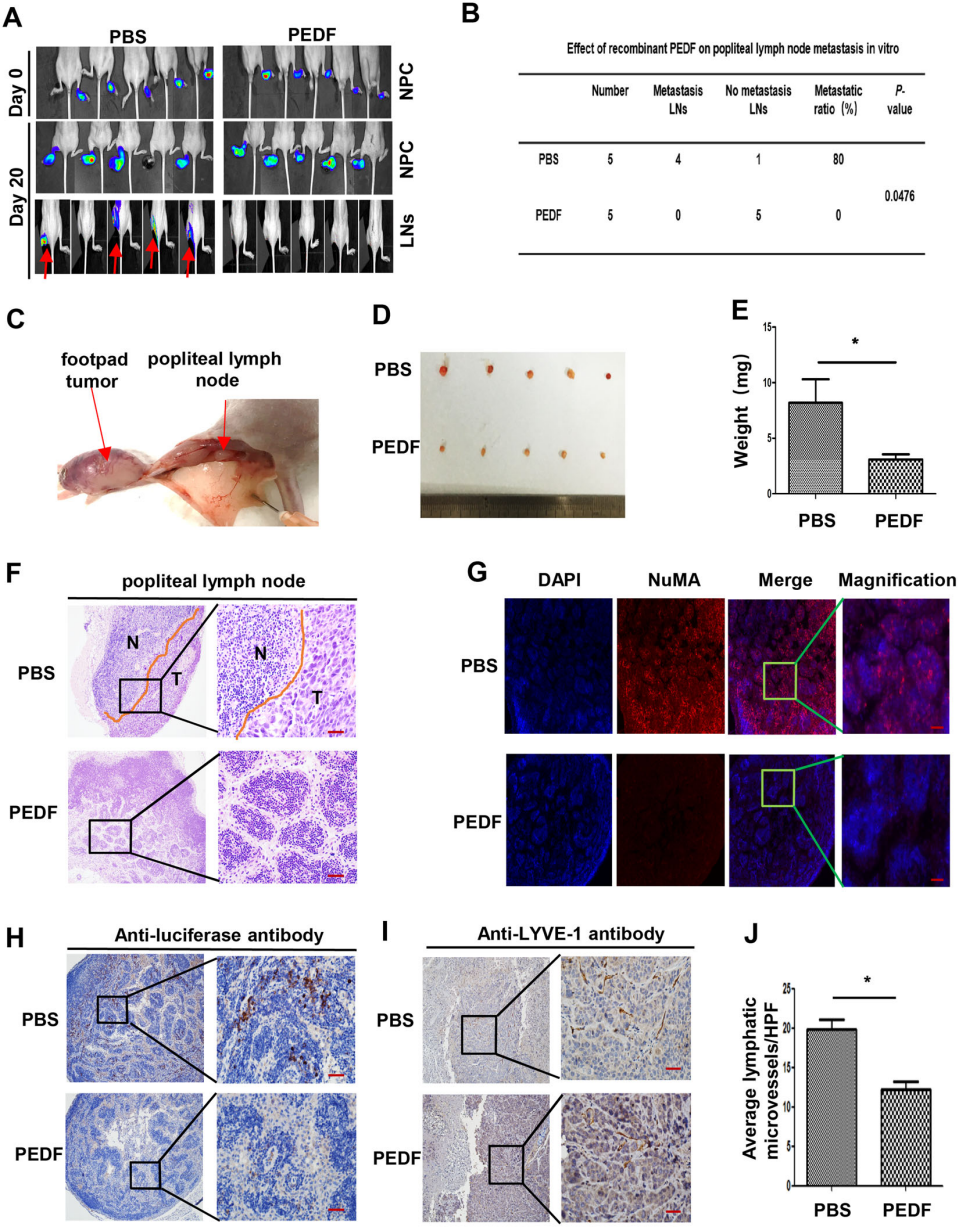
Recombinant PEDF reduces the LVD and lymph node metastasis in the cell-derived xenograft animal model. **A** Image of the nasopharyngeal cancer xenografts and homolateral popliteal lymph nodes in nude mice treated with PBS or recombinant PEDF for 20 days. The red arrows mean the metastasized cancer cells. **B** The table shows the statistical results of the metastatic ratio in these two groups. **C**–**E** The representative morphology of the foot-pad tumors and homolateral popliteal lymph nodes in nude mice (**C**), micrographs of separated popliteal lymph nodes (**D**), and lymph nodes weight (**E**). **F** H&E staining of homolateral popliteal lymph nodes and the orange dotted line represents the boundary between metastatic tumors and normal lymph nodes (image magnification, 200×). **G** The homolateral popliteal lymph nodes were stained with NuMA (image magnification, 400×) by IF and luciferase (image magnification, 200×) by IHC (**H**) to detect metastatic cancer cells. **I** LVD in the nasopharyngeal cancer xenografts, lymphatic tubes were stained with LYVE-1 (image magnification, 200×), and the statistical result of LVD in the PBS group and PEDF group (**J**). *n* = 5, Scale bars:100 μm (**F**, **H**, **I**), 50 μm (**G**), *p < 0.05, the results are presented as mean ± SD.

It is reported that some angiogenesis inhibitors can also inhibit lymphangiogenesis. The above study showed that PEDF had a negative correlation with LVD and lymph node stage in human NPC tissues, which suggested that PEDF might have the potential to inhibit lymphangiogenesis. In order to confirm the relation between PEDF and lymph node metastasis, we constructed a model of the plantar popliteal lymph node metastasis by inoculated S18 cells, which stably express firefly luciferase, into the foot-pad of nude mice. After 20 days of 640 nM recombinant PEDF (rPEDF) or PBS treatment, the in vivo imaging showed no significant metastases in a popliteal lymph node in the rPEDF-treated group on day 20, while four mice in the PBS group had metastases (*n* = 5/group, Fig. 2A, B). After the mice were sacrificed, the popliteal lymph nodes were enucleated. The weight of popliteal lymph nodes in the PBS group was more than the rPEDF group (Fig. 2C–E). The metastasized NPC cells in the popliteal lymph nodes were detected by H&E staining and immunofluorescence with NuMA or luciferase antibody, which could specifically recognize inoculated human NPC cells. After 20 days of PEDF treatment, there were few NPC cells in the homolateral popliteal lymph nodes, while the PBS group had evident metastasis (Fig. 2F–H).

To further explore the role of PEDF on lymphangiogenesis in NPC xenografts, we stained the lymphatic vessels in the tumor by IHC, and the results revealed that the lymphatic vessels in the PEDF-treated group were significantly reduced compared with the PBS group (Fig. 2I, J). These findings revealed that PEDF reduced the LVD and lymph node metastasis in vivo.

### PEDF inhibits proliferation, migration, tube formation, and promotes apoptosis of lymphatic endothelial cells

**Fig. 3:**
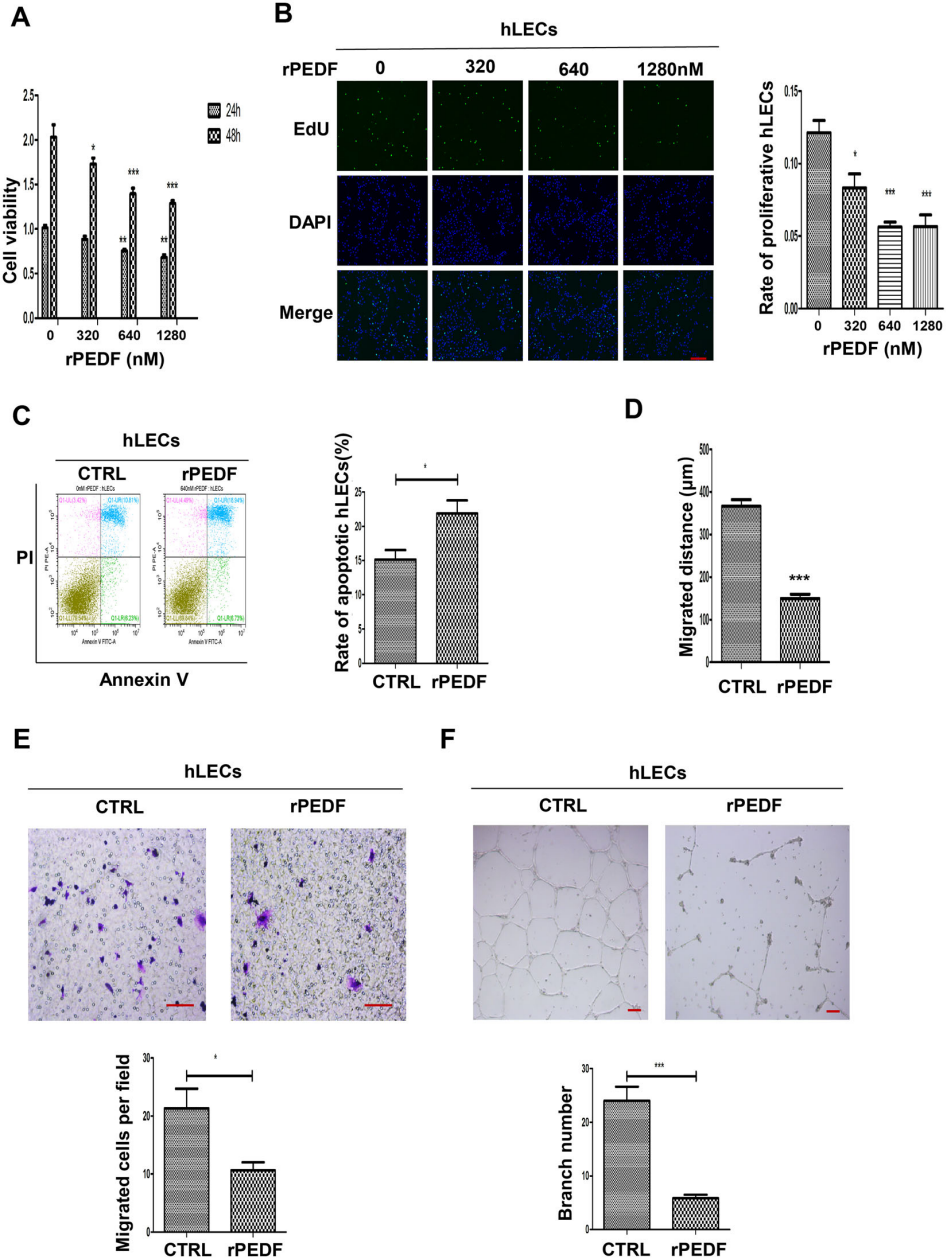
PEDF inhibits proliferation, migration, tube formation, and promotes apoptosis of lymphatic endothelial cells. **A**, **B** The proliferation of hLECs was examined by CCK8 assay (**A**) and EdU (**B**) after BSA or rPEDF treatment for 48 h (image magnification, 100×). The histogram represents the rate of proliferative hLECs. **C** The apoptotic hLECs were quantified by flow cytometry after incubation with BSA or 640 nM rPEDF for 48 h. The histogram represents the rate of apoptotic hLECs. **D**, **E** The migration of hLECs was measured with the cell scratch test (**D**) and transwell migration assay (**E**) after BSA or 640 nM rPEDF treatment for 12 h (image magnification, 200×). The histogram represents migrated cells per field. **F** Tube formation assay was used to test the lymphangiogenesis capacity in hLECs after BSA or 640 nM rPEDF treatment for 12 h (image magnification, 100×). The histogram represents the branch number per field. Scale bars: 100 μm (**B**, **E**, **F**), *p < 0.05, ***p < 0.001, the results are presented as mean ± SD.

To detect the mechanism by which PEDF inhibits lymphangiogenesis, we conducted some experiments to confirm whether PEDF had direct effects on lymphatic endothelial cells (hLECs). hLECs were treated with 0, 320, 640, and 1280 nM recombinant PEDF protein (rPEDF) for 24 h and 48 h, respectively. CCK8 results showed that 640 and 1280 nM rPEDF had obvious inhibitory effects on hLECs growth after treatment for 24 h. After 48 h of treatment, 320 nM rPEDF also inhibited the proliferation of hLECs (Fig. 3A). The EdU assay showed that 640 and 1280 nM PEDF treatment significantly inhibited the proliferation of hLECs (Fig. 3B). Subsequently, we examined the effect of PEDF on the apoptosis of hLECs, and the apoptotic rate of hLECs was increased significantly after treatment with PEDF for 48 h (Fig. 3C).

Furthermore, the scratch test showed that 640 nM recombinant PEDF on hLECs for 12 h significantly decreased the migration distance of hLECs than the BSA-treated group (Fig. 3D). The transwell chamber results showed that the number of hLECs from the upper chamber to the lower chamber was significantly reduced after PEDF treatment for 12 h (Fig. 3E). In addition, the tube formation assay showed that PEDF decreased the tube formation of hLECs (Fig. 3F). In summary, these findings indicated that PEDF inhibited proliferation, migration, tube formation, and promoted apoptosis of lymphatic endothelial cells.

### PEDF is negatively correlated with VEGF-C in NPC tissues and cell lines and inhibits the expression of VEGF-C

**Fig. 4:**
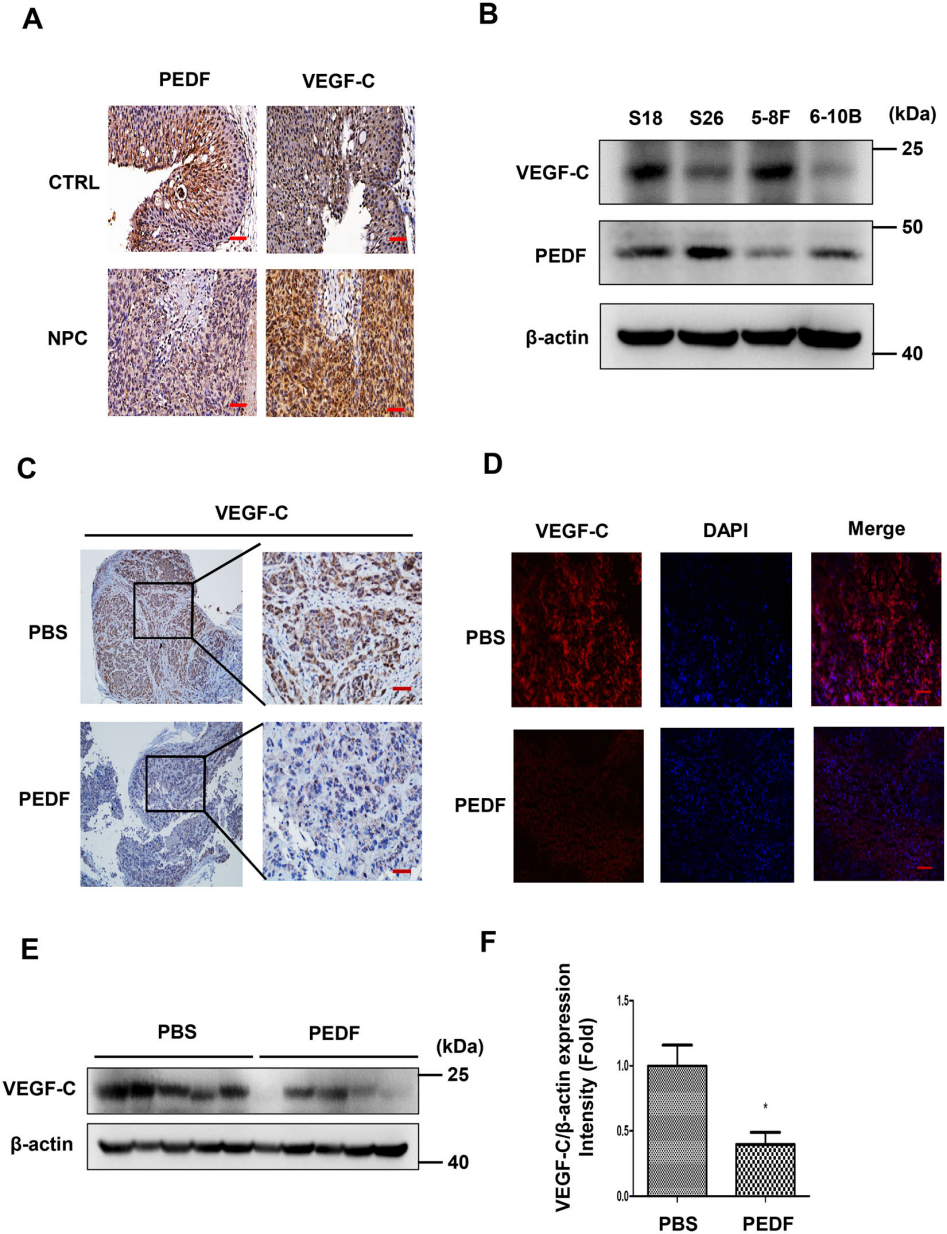
PEDF was negatively correlated with VEGF-C in NPC tissues and cell lines and could inhibit the expression of VEGF-C in vivo. **A** VEGF-C staining in tissue microarray by IHC (image magnification, 200×). **B** The PEDF and VEGF-C expression in different nasopharyngeal cancer cell lines, S18, S26, 5–8 F, and 6–10B. **C**–**F** VEGF-C staining (image magnification, 200×) by IHC (**C**), IF (**D**), and western blot (**E**, each lane represents the sample from an individual mouse) in nasopharyngeal cancer xenografts treated with PBS or rPEDF for 20 days, and the expression of VEGF-C was quantified by grayscale scanning with Image J (**F**). Scale bars:100 μm (**A**, **C**, **D**), *p < 0.05, the result is presented as mean ± SD.

VEGF-C is a crucial regulator of lymphangiogenesis by binding to VEGFR-3. It could induce vascular permeability and stimulate proliferation and migration of lymphatic endothelial cells, and VEGF-C secreted from tumors plays a vital role in lymphangiogenesis^35,36^. To further explore the correlation between PEDF and VEGF-C, we detected the VEGF-C expression in NPC tissues, xenografts, and several NPC cells. The results showed that the VEGF-C level in NPC tissues was upregulated with PEDF downregulation in a NPC tissue microarray (Fig. 4A). Cell experiments showed that the expression of PEDF in these cells was negatively correlated with the expression of VEGF-C (Fig. 4B).

**Fig. 5:**
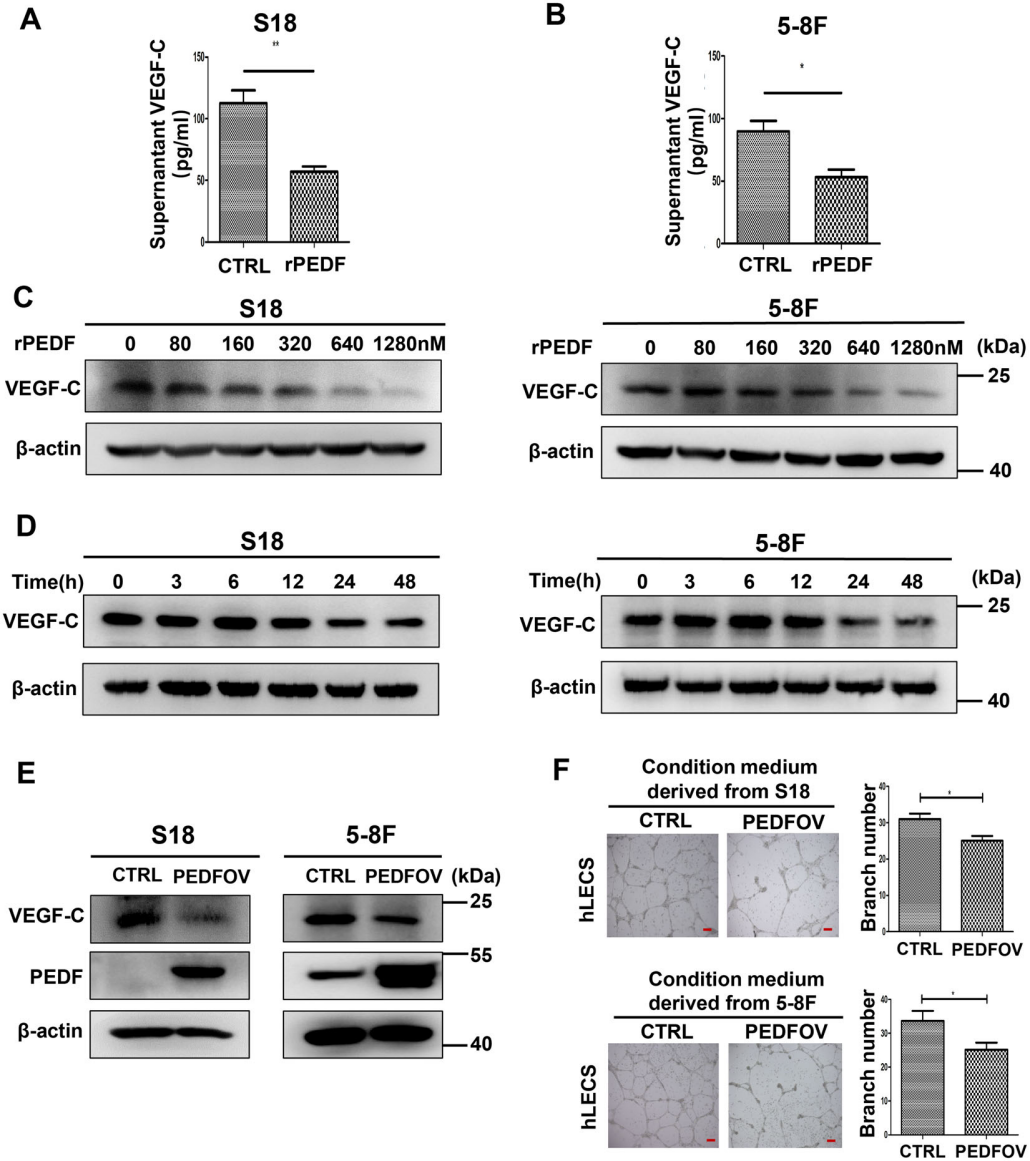
PEDF inhibits the expression and secretion of VEGF-C in nasopharyngeal carcinoma cells. **A**, **B** The VEGF-C concentration of (**A**) S18 and (**B**) 5–8 F cells in the cell culture medium was detected by ELISA after BSA and 640 nM recombinant PEDF treatment for 48 h. **C** VEGF-C expression in S18, S26, 5–8 F, and 6–10B cells treated with different doses of rPEDF for 48 h, was detected by western blot. **D** VEGF-C expression in S18 and 5–8 F cells treated with 640 nM rPEDF for 0, 3, 6, 12, 24, and 48 h was detected by western blot. **E** S18 and 5–8 F cells were transfected with PEDF plasmid or vehicle plasmid for 48 h. PEDF and VEGF-C protein levels were measured by western blot. **F** Representative images of Matrigel tube formation assay in hLECs were cultured with conditioned medium derived from control NPC cells and PEDF overexpressing NPC cells (image magnification, 100×). Scale bars:100 μm (**F**), *p < 0.05, **p < 0.01, the results are presented as mean ± SD.

We next aimed to confirm the effect of PEDF on the expression and secretion of VEGF-C in vitro. In the mice xenografts, PEDF treatment decreased VEGF-C expression in the tumor tissues than the PBS group with IHC (Fig. 4C), immunofluorescence (Fig. 4D), and western blot (Fig. 4E, F). In vitro study, S18, 5–8 F cells were treated with 640 nM rPEDF for 48 h, and the concentration of VEGF-C in the cell culture medium in the PEDF group was lower than that in the BSA group (Fig. 5A, B), which indicated that PEDF downregulated VEGF-C secretion in NPC cells. PEDF also exhibited a concentration-dependent and time-dependent inhibition of VEGF-C expression (Fig. 5C, D and Supplementary Fig. S1A–D). Similarly, overexpression of PEDF in S18 and 5–8 F cells decreased the expression of VEGF-C (Fig. 5E and Supplementary Fig. S1E, F). Furthermore, tube formation assay in hLECs with conditioned medium derived from PEDF overexpression NPC cells inhibited the tube formation of lymphatic endothelial cells (Fig. 5F). Taken together, these results suggested that PEDF inhibited the expression and secretion of VEGF-C in NPC tissues and cell lines.

### PEDF downregulates the expression of VEGF-C through IKK/IҡB/NF-ҡB signaling pathway in NPC cells

**Fig. 6:**
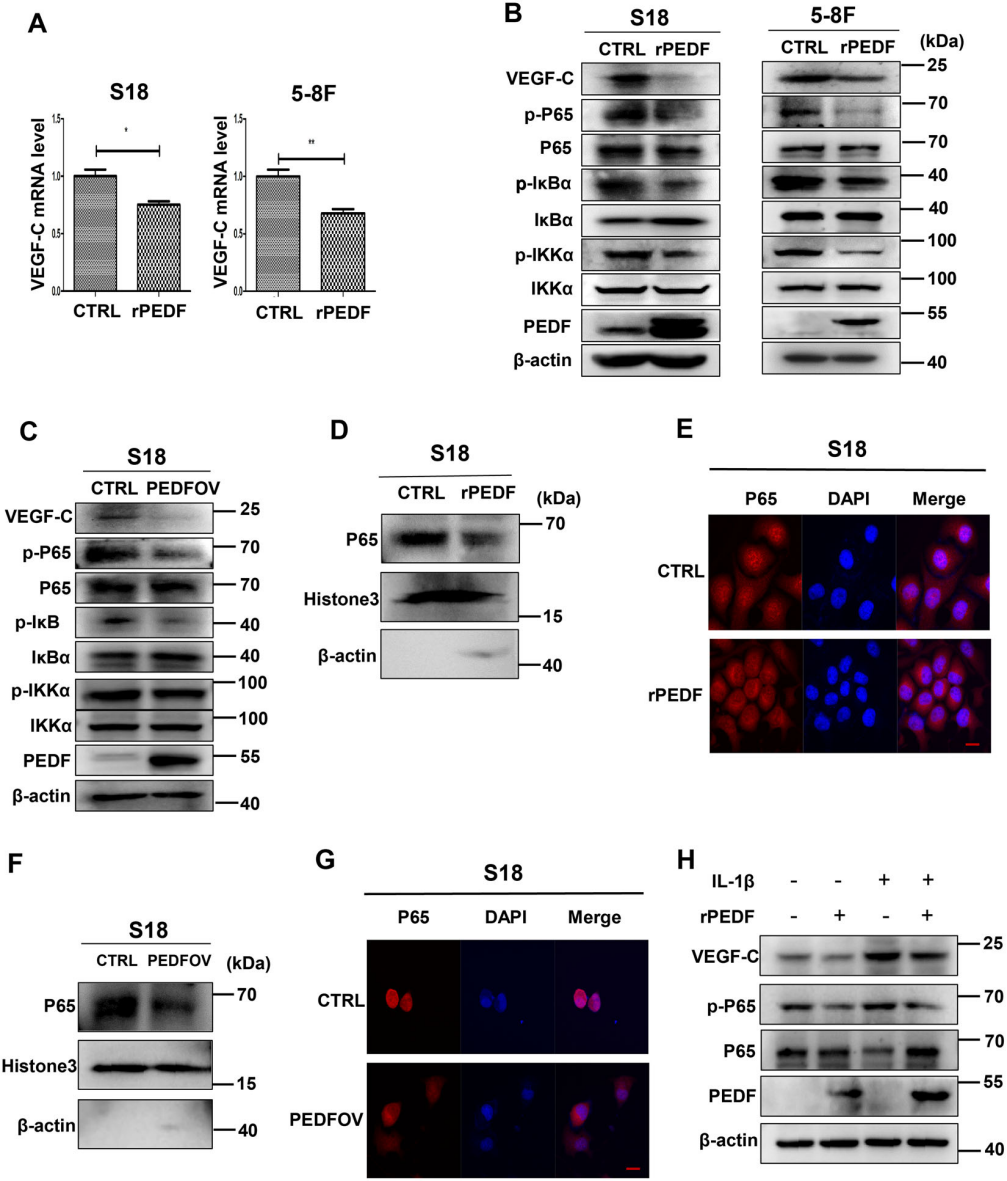
PEDF downregulates the expression of VEGF-C through IKK/IҡB/NF-ҡB signaling pathway in nasopharyngeal carcinoma cells. **A** The VEGF-C mRNA levels of S18 and 5–8 F cells were detected by qRT-PCR after BSA and 640 nM recombinant PEDF treatment for 48 h. **B**, **C** NF- ҡB pathway proteins in S18 and 5–8 F cells incubated with BSA or 640 nM rPEDF (**B**) and transfected with PEDF plasmid for 48 h (**C**) were measured by western blot. **D**, **G** The nuclear p-P65 protein level was tested by western blot, and P65 nucleus translocation (image magnification, 1000×) was detected by immunofluorescence in S18 cells after BSA or 640 nM rPEDF treatment (**D**, **E**) and PEDF plasmid (**F**, **G**) for 48 h (image magnification, 1000×). **H** The protein levels of VEGF-C and p-P65 in S18 cells incubated with BSA, 640 nM rPEDF, NF-ҡB activator IL-1β, IL-1β + 640 nM rPEDF for 48 h. Scale bars:20 μm (**E**, **G**), *p < 0.05, **p < 0.01, the results are presented as mean ± SD.

Previous studies reported that the NF-ҡB signaling pathway was involved in the regulation of VEGF-C expression, and P65 was showed to be a transcript factor for VEGF-C^37–39^. Here we tested whether PEDF regulates VEGF-C expression through inhibiting NF-ҡB activation. S18, 5–8 F cells were treated with 640 nM rPEDF or BSA for 48 h, and the transcription level of VEGF-C was decreased in the rPEDF group compared with the BSA group (Fig. 6A). Subsequently, we detected its effect on the NF-ҡB signaling pathway. The results showed that rPEDF and PEDF overexpression inhibited the phosphorylation of IKKα and IҡBα, followed by the p-P65 decrease, which led to a decreased level of VEGF-C (Fig. 6B, C and Supplementary Fig. S1G–I). In addition, the translocation of P65 was inhibited by rPEDF treatment (Fig. 6D, E and Supplementary Fig. S1J) and PEDF overexpression (Fig. 6F, G and Supplementary Fig. S1K). Next, NF-ҡB activator IL-1β was applied to rescue the inhibition of PEDF on nuclear factor-κB (NF-κB). Thus the expression of VEGF-C was recovered (Fig. 6H and Supplementary Fig. S1L). These findings suggested that PEDF downregulated VEGF-C expression in the transcription level through inhibiting the NF-ҡB signaling pathway.

**Fig. 7:**
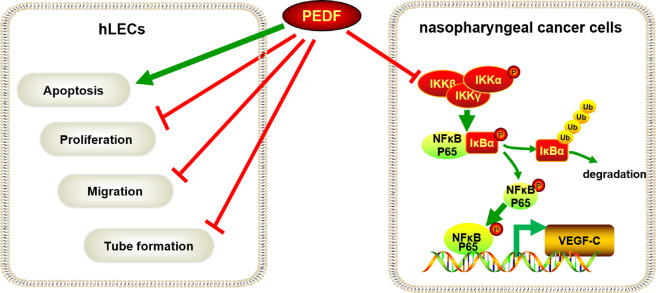
The illustrative model of PEDF in lymphangiogenesis of NPC. The illustrative model shows the proposed mechanism by which PEDF inhibits lymphangiogenesis and lymphatic metastasis in NPC via downregulation of VEGF-C expression in nasopharyngeal cancer cells and inhibits the proliferation, migration, tube formation of hLECs, and promote its apoptosis.

In a word, our results indicate that PEDF directly inhibits lymphatic endothelial cell proliferation, migration, tube formation and promotes cell apoptosis in vitro. PEDF also inhibits NPC cells from expressing and secreting VEGF-C through the NF-κB signaling pathway. Low expression of PEDF thereby induces lymphangiogenesis and lymphatic metastasis and leads to poor prognosis in NPC patients (Fig. 7).

## Discussion

Previous studies have shown that the function of PEDF mainly includes anti-angiogenesis, neuroprotection, and regulating lipid metabolism, but no one has studied its relationship with lymphangiogenesis and lymphatic metastasis in tumors^32,33^. Our study demonstrated that PEDF was lowly expressed in NPC tissues with poor prognosis and negatively correlated with lymphatic vessel density (LVD). Further experiments showed that PEDF directly inhibited lymphatic endothelial cell proliferation, migration, tube formation, and promoted cell apoptosis in vitro. PEDF also reduced the expression and secretion of VEGF-C through the NF-κB signaling pathway in NPC cells. It proves for the first time that PEDF is also a potent endogenous lymphangiogenesis inhibitor.

Lymphatic metastasis is a dominant way of metastasis and leads to poor prognosis in NPC, but there is no proper clinical treatment for lymphatic metastasis. A large number of studies have shown that lymphangiogenesis plays a significant role in cancer lymphatic metastasis, including melanoma and many tumors of epithelial origin, such as breast cancer and gastric cancer, and the inhibition of tumor-induced lymphangiogenesis could reduce the distant metastasis of these cancers^9,40^. The lymphangiogenesis process is mainly the migration of lymphatic endothelium from the existing lymphatic vessels and the formation of new lymphatic vessels^10^. Only very few drugs specifically designed to block this process are in clinical testing, mainly because the research on tumor lymphangiogenesis and its role in the metastatic process is infancy.

These drugs are mainly small molecular inhibitors or antibodies of VEGF-C, VEGFR-3, and c-MET^41^. Based on the previous disadvantages in angiogenesis inhibition, these drugs are prone to produce drug resistance with unreliable specificity and noticeable side effects. In addition, these antagonists are single-target drugs but have no function on other pro-lymphangiogenesis factors, so the effect is limited. The first newly discovered lymphangiogenesis inhibitor, sVEGFR-2, can only inhibit lymphangiogenesis but does not affect angiogenesis, which will restrict its use^26^. On the other hand, there are many deficiencies in endogenous lymphangiogenesis inhibitors. Endostatin only inhibits lymphatic neoplasm by inhibiting the secretion of VEGF-C of tumor cells^22^. Thrombospondin-1 (TSP-1), a potent inhibitor of angiogenesis that predominantly acts through its receptor CD36, has been reported to inhibit inflammatory lymphangiogenesis, whereas TSP-1 did not inhibit tumor-induced lymphangiogenesis in a chemical skin carcinogenesis model^24,25^. Therefore, it becomes imperative to look for new drugs targeting tumor lymphangiogenesis in NPC.

The endogenous angiogenesis inhibitor PEDF, a member of the serine protease inhibitor (Serpin) family, has attracted our attention because it targets proliferate vascular endothelial cells and has no effect on the existing healthy blood vessels with marginal side effects and rare drug resistance^32^. Our research showed that PEDF was lowly expressed in NPC tissues and negatively correlated with LVD. The GEO database analysis results also showed that PEDF mRNA in NPC was lower than adjacent tissues (NCBI/GEO/GSE53819, *p* < 0.001) (Supplementary Fig. S2). In contrast, there was no significant correlation between PEDF and patient outcomes in 5-years survival. It may be due to the low proportion of patients with positive PEDF expression (only 8.9%; 15/168) and the high rate of 5-years survival in NPC patients (69.0%, 116/168) (Supplementary Fig. S3). Meanwhile, we found that head and neck squamous cell carcinoma with high PEDF expression had a more prolonged overall survival in the GEO database (*P* = 0.049, HR = 0.6) (Supplementary Fig. S4).

Our study also found that PEDF could directly inhibit proliferation, migration, tube formation, and promote apoptosis of hLECs. Previous studies have shown that the MAPK signaling pathway plays an essential role in the lymphangiogenesis of lymphatic endothelial cells, and activation of P38 and ERK is thought to induce its proliferation and tube formation^37,42^. Our preliminary results revealed that PEDF inhibited MAPK signaling and downregulated the expression of ZO-1, VE-cadherin (cell junction marker), MLC2, Paxillin (migration marker), and p-RB, p-4E-BP1, cyclin D1 (proliferation marker) in hLECs (Supplementary Fig. S5), the detailed mechanisms are worthy of being explored in the future. These suggest that in addition to anti-angiogenesis, PEDF also inhibits lymphangiogenesis, which may better affect tumor metastasis.

It was previously reported that the expression of VEGF-C was higher in NPC patients with lymphatic metastasis^43^. We found that PEDF was low in NPC and was negatively correlated with the LVD and VEGF-C in NPC tissues. GEO database analysis also revealed that PEDF was negatively correlated with VEGF-C in NPC and head and neck squamous cell carcinoma (Supplementary Fig. S6A, B). VEGF-C is the most crucial inducer in lymphangiogenesis, and its interaction with VEGFR3 is necessary for lymphangiogenesis, so it is significant to regulate VEGF-C^36^. Our experiments have found that PEDF not only inhibits hLECs proliferation, migration, tube formation, and promotes its apoptosis but also inhibits the expression and secretion of VEGF-C in NPC cells. It has been reported that NF-κB P65 is a direct transcription factor to VEGF-C. NF-κB signaling influences a broad range of biological processes, including innate and adaptive immunity, inflammation, stress responses, B-cell development, and lymphoid organogenesis^44,45^. Our research makes clear that PEDF reduces the activation of IKKα and the phosphorylation of IκBα, which leads to the degradation of IκBα. Finally, the weakened translocation of P65 results in the downregulated VEGF-C expression. Moreover, the luciferase assay and CHIP assay confirmed the detachment of p65 from the VEGF promoter upon PEDF overexpression (Supplementary Fig. S7). Therefore, on the one hand, PEDF, as an endogenous inhibitor in angiogenesis, directly acts on lymphatic endothelial cell proliferation, migration, tube formation. On the other hand, PEDF also reduces VEGF-C expression and secretion of tumor cells. The dual effects together inhibit the lymph node metastasis of NPC.

The study of endogenous lymphangiogenesis inhibitors will further help us understand the regulation of lymphangiogenesis and help us find the treatment for lymphatic metastasis. Although the mechanism of PEDF for lymphatic endothelial cells and tumor cells needs further exploration, our results have provided strong evidence that PEDF is a potent endogenous lymphangiogenesis inhibitor and plays a crucial role in nasopharyngeal lymphangiogenesis and lymphatic metastasis, which will provide a candidate drug for the treatment of NPC metastasis.

## Material and methods

### Cell lines and cell culture

Human NPC cell lines S18, S26, 5–8 F, 6–10B were gifted by professor Chaonan Qian from Sun Yat-sen University Cancer Center. Human lymphatic endothelial cells (hLECs) were purchased from Procell (Wuhan, China). The NPC cell lines were cultured with 10% FBS DMEM medium, and hLECs (<6 passages) were cultured in ECM medium (ScienCell, San Diego, CA). They were all incubated in a humidified incubator at 5% CO_2_ and 37 °C.

### Antibodies and reagents

Antibodies for VEGF-C, P65, p-P65, IκBα, p-IκBα, IKKα, p-IKKα, P38, p-P38, JNK, and p-JNK were purchased from Cell Signaling Technology (Danvers, MA, USA), Antibodies for p-ERK (E4) and ERK 1 (K-23) from Santa Cruz Biotechnology (Santa Cruz, CA, USA), LYVE-1, histone 3, and D2-40 from Abcam (Cambridge, Massachusetts, USA) and β-actin from Sigma-Aldrich (Saint Louis, Missouri, USA), PEDF antibody from Millipore (Bedford, MA, USA), and IL-1β recombinant protein were from Sigma-Aldrich.

### Human NPC tissue microarrays and clinical samples

The NPC tissue microarrays were purchased from Auragene Bioscience (Changsha, China), which contained human nasopharyngeal cancer (22 cases), papilloma (15 cases), polyp (6 cases), hyperplasia, and inflammation (6 cases) tissues. The tissue samples of NPC (*n* = 168) were provided by Sun Yat-Sen University Cancer Center (Guangzhou, China). Inclusion criteria were that patients did not receive chemotherapy or radiotherapy before the operation. The data of clinicopathological parameters were obtained from patients’ clinical records and pathological reports. All patients’ informed consent was obtained before surgery. This study was in accordance with the principles outlined in the declaration of Helsinki, approved by the medical ethics committee of Sun Yat-Sen University.

### Experimental animals and popliteal lymph node metastasis model

Male BALB/c nude mice (5–6 weeks old, 20–22 g) were obtained from Vital River(Beijing, China). The mice were kept in a specific pathogen-free (SPF) condition with a 12-hours light/dark cycle, and the humidity and temperature were controlled at 40–70% and 22 ± 3 °C. After 1 week of adaptive feeding, the NPC line S18 cells (1 × 10^6^), which were infected with the pLenti-CMV-EGFP-linker-Luc-PGK-Puro virus (Obio, Shanghai, China) previously, were inoculated subcutaneously into the foot-pads of BALB/c nude mice to generate a primary tumor. After inoculation for 1 week, the nude mice were randomly divided into two groups according to the method of random number table. The rPEDF-treated group was injected intraperitoneally with 50 mg/kg body weight recombinant PEDF (1000 nM) in 48 h intervals, and the control group was injected with the same volume of PBS. During the treatment, the nude mice’s body weight, length, and width of the tumors were measured, then the volume was calculated. Tumor volume is calculated by the following formula: Volume (mm^3^) = (length × width ^2^) / 2. After 20 days of treatment, in vivo imaging of plantar implants and popliteal lymph nodes were achieved by IVIS Spectrum Imaging System (PerkinElmer) after injection of luciferin (Promega). Mice were then sacrificed and weighed, the implanted tumors and popliteal lymph nodes were dissected and weighed, and some were used for western blotting detection and paraffin-embedding. Measurements and outcome assessments were performed by a researcher who was blinded to group allocation. All the animal experiments were conducted in accordance with the Guidelines for the Use of Laboratory Animal at Sun Yat-Sen University.

### Expression and purification of recombinant PEDF

The expression and purification of recombinant PEDF were performed as described before, and all details were in our previous articles^46^.

### qRT-PCR

Total RNA was extracted from NPC cell lines using a total RNA extraction kit (QIAGEN, Beijing, China). RNA was reverse-transcribed using Takara’s PrimeScript Reverse Transcription Kit, and then Takara’s SYBR II Premix Ex TaqTM was used for the qPCR reaction system. Finally, Roche’s LightCycler 2.0 system was used for real-time quantitative PCR analysis. The sequences of PCR primers used for amplification of VEGF-C and beta-actin were as follows:human VEGF-C (sense primer: 5′-CTCAAGGCCCCAAACCAGTA-3′, antisense primer: 5′-GCCTGACACTGTGGTAGTGTT-3′). β-actin (sense primer: 5′-ACTCTTCCAGCCTTCCTTC-3′, antisense primer: 5′-ATCTCCTTCTGCATCCTGTC-3′). The above primers were all synthesized by GeneRay(Guangzhou, China). β-actin was used as an internal control, and all samples were assayed in triplicate.

### Cell viability assay

hLECs were seeded in 96-well plates at 5000 cells per well. After the cells attached to the plates, the culture medium was replaced with a serum‐free medium, and then cells were treated with different concentrations of recombinant PEDF or BSA for 24 and 48 h. Cell viability was measured using Cell Counting Kit-8 (DOJINDO, Japan). The absorbance value at 450 nm was assessed using a Sunrise Microplate Reader (TECAN, AG, Switzerland).

### EdU staining

EdU staining was performed according to the protocol of the EdU staining kit (KeyGEN, Nanjing, China), then sections were visualized with ZEISS Axio Imager Z1 (ZEISS, Jena, Germany).

### Wound healing assay

hLECs were seeded in 6-well plates and then was scratched with a 10 µl pipette tip to obtain scratches of a constant width when cells reached 100% confluence. Cells were then incubated with the rPEDF treatments, and cells invading the wound line after 12 h were photographed with ZEISS Axio Imager Z1 (ZEISS, Jena, Germany).

### Tube formation assay

The hLECs tube formation assay was performed by pipetting 200 μL Matrigel (BD Biosciences, Bedford, Massachusetts, USA) into each well of a 24-well plate firstly. After Matrigel polymerized for 30 min at 37 °C, hLECs (2 × 10^4^) were added to each well with 200 μL of conditioned medium and incubated at 37 °C, 5% CO_2_ for 12 h. Images were photographed with ZEISS Axio Observer Z1 (ZEISS, Jena, Germany).

### Apoptosis assay

Apoptosis assay was performed using flow cytometry with a commercial Annexin V-FITC Apoptosis Kit (Vazyme Biotech, Nanjing, China) according to the manufacturer’s protocol. The hLECs were treated with 640 nM rPEDF for 48 h, and then the cells were harvested according to the manufacturer’s recommendations. Subsequently, cells were re-suspended with binding buffer containing Annexin V-FITC and propidium iodide (PI), then go for analysis on a Beckman CytoFLEX flow cytometer (Beckman Colter, CA, USA) after a 15-minutes incubation at room temperature in the dark.

### Immunohistochemistry

Tissues from mice were embedded in paraffin, and 5 μm thick histologic sections were prepared for IHC analysis. After antigen retrieval of paraffin sections, endogenous peroxidase blocking solution and normal goat serum were used to block non-specific background. Sections were then incubated with indicated antibodies at 4 °C overnight, and then the slides were incubated with a biotin‐conjugated secondary antibody (DAKO, Glostrup, Denmark) for 60 min at room temperature, followed by counterstaining with hematoxylin. Images of paraffin sections and tissue microarrays were obtained by Pannoramic Viewer software, and the quantification of protein expression level was assessed by grayscale scanning with Image pro-plus. Briefly, all the IHC photos were taken under the same microscope environment and shooting conditions. The first step of the analysis was correcting the optical density. After deducting the background value, the correction was applied to all images as the optical density value system. After that, the color was selected using HSI mode in segmentation, select H: 0–30, S: 0–255, I: 0–230, and fine adjustment was made according to the picture situation, then the color selection settings were saved. The third step was setting the analysis environment of the software and saving the analysis environment. Finally, the value of IOD SUM was measured and read as the integrated optical density of this image. Every specimen has two spots in this tissue microassay, and the average IOD SUM of the two spots was taken as the total expression of the specimen. The malignant tumor group included human nasopharyngeal cancer (22 cases). The others (27 cases), including papilloma (15 cases), polyp (6 cases), hyperplasia, and inflammation (6 cases), were the control group. Student’s *t* test analysis was performed to compare the statistical data between two groups.

### Western blotting

Proteins were separated by SDS-PAGE and transferred onto PVDF membrane. Membranes were incubated in 7% nonfat dry milk, followed by incubation with primary antibodies for 12–18 h and second antibody for 4 h at 4 °C. After washing 3 times, membranes were developed using Clarity Western ECL Substrate according to the manufacturer’s instructions.

### Enzyme-linked immunosorbent assay

VEGF-C in the culture supernatants of tumor cells were quantified using human VEGF-C ELISA Kit (R&D Systems, USA) according to the manufacturer’s protocol.

### Nuclear Protein Extraction

S18 cells were treated with 640 nM rPEDF or BSA and transfected with PEDF plasmid or control vector for 48 h before the extraction of total protein and then extracted nucleoprotein using a Nucleoprotein Extraction Kit (Thermo). The operation procedures were carried out in strict accordance with the instructions of the kit.

### Immunofluorescent staining

Cells were treated with 640 nM rPEDF for 48 h then were fixed in 4% (w/v) paraformaldehyde, permeabilized with 0.1% (v/v) Triton X-100 in PBS, and stained with a 1:200 dilution of rabbit anti-p65 polyclonal antibody (CST, Danvers, MA, USA). After washing, the primary antibody was visualized with rhodamine-conjugated goat anti-rabbit IgG, and the cell nucleus was stained with DAPI. P65 in the nucleus was observed using a confocal microscope (Carl Zeiss, Oberkochen, Germany).

### Bioinformatics mining

Datasets (GSE53819) from GEO (https://www.ncbi.nlm.nih.gov/gds) were mined to predict the PEDF differential expression level in NPC adjacent tissues and cancer tissues groups. PEDF and VEGF-C correlation analyses conducted by Pearson correlation statistics were carried out with GEO and GEPIA (http://gepia.cancer-pku.cn/) for all the given sets of TCGA expression data. The survival analyses of HNSCC, performed by GEPIA for hypothesis evaluation, used a log-rank test based on gene expression levels.

### Statistical analysis

Statistical analysis was performed using the SPSS 13.0 software. Power calculations were conducted to determine appropriate sample size, Student’s *t* test analysis was performed to compare the statistical data between two experimental groups, and one-way ANOVA was used to analyze the difference in data between groups (ANOVA, one-way analysis of variance), followed by the least significant difference test (LSD-t) if the variance is similar between the groups, otherwise the Games–Howell method is required. The *χ*2 test was used to analyze the relationships between PEDF expression and clinicopathological characteristics. The Spearman correlation test (2-tailed) was used to calculate the correlation coefficient (*r*) and *P* value between the LVD and grayscale value of PEDF staining. All data meet the assumptions of the statistical test used. All data are presented as mean ± SD. The test level was 0.05. *P* values less than 0.05 were defined as statistically significant.

## Supplementary information

supplementary data

Supplementary Table 1-editable
