# Supplementary material for: PEDF inhibits lymphatic metastasis of nasopharyngeal carcinoma as a new lymphangiogenesis inhibitor
Source: Cell Death Dis. 2021 Mar 17;12(4):295. doi: 10.1038/s41419-021-03583-1 (PMC7969934; doi:10.1038/s41419-021-03583-1)
Supplement: Supplementary file 1 — supplementary data [file 41419_2021_3583_MOESM1_ESM.pdf]

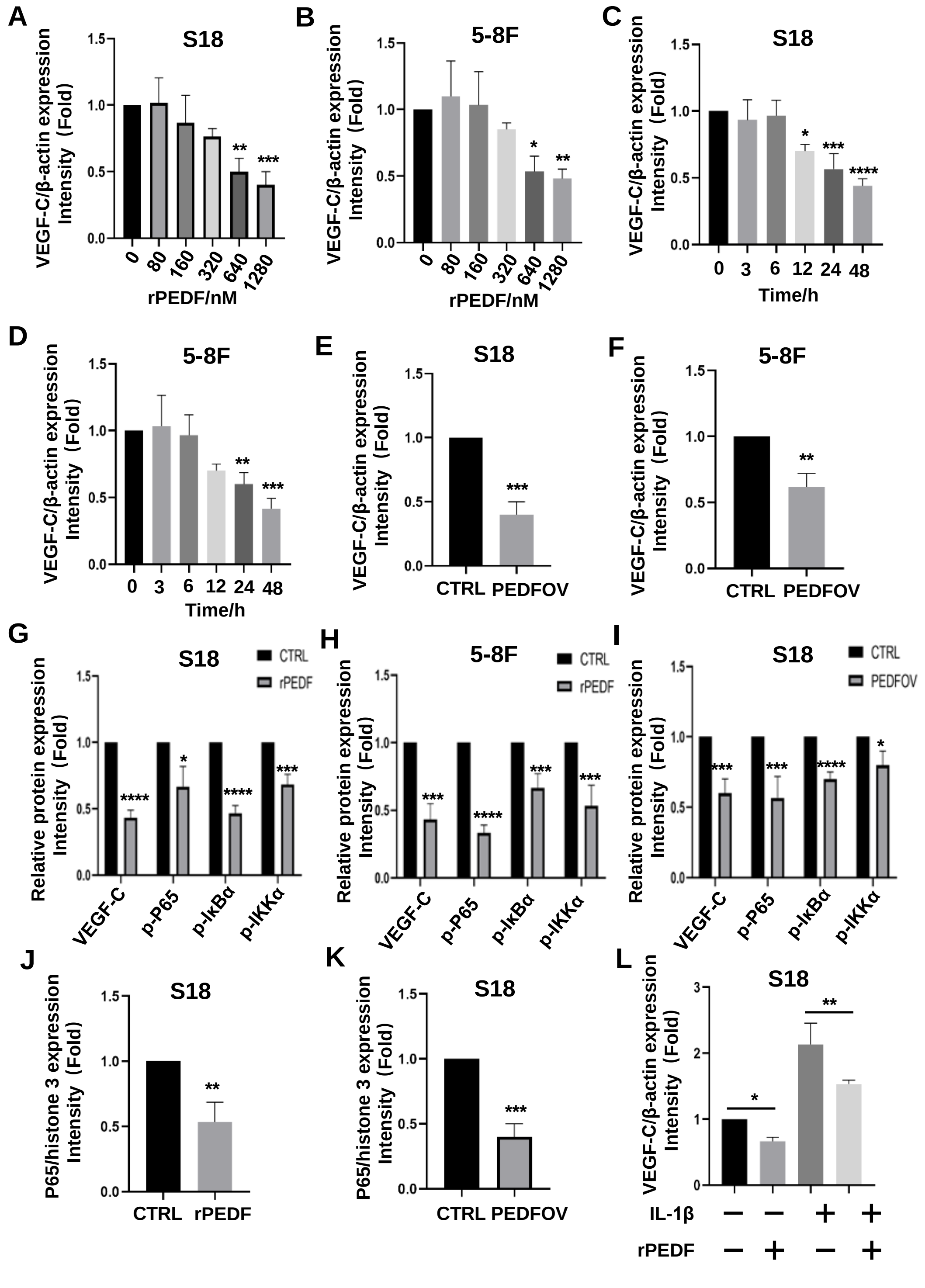
A
S18
VEGF-C/β-actin expression Intensity (Fold)
rPEDF/nM
B
5-8F
C
S18
Time/h
D
5-8F
E
S18
CTRL
PEDFOV
F
5-8F
G
S18
CTRL
rPEDF
Relative protein expression Intensity (Fold)
VEGF-C
p-P65
p-IκBα
p-IKKα
H
5-8F
I
S18
J
S18
P65/histone 3 expression Intensity (Fold)
K
S18
L
S18
IL-1β
rPEDF

Supplementary Figure 1. Densitometric analysis of western blot data in figure 5 and 6. (A-F) The densitometric analysis of figure 5C-E, (G-L) The densitometric analysis of figure 6B&C&D&F&H, respectively.*$p < 0.05$, **$p < 0.01$,*** $p < 0.001$, ,**** $p < 0.001$, the results are presented as mean ± SD.

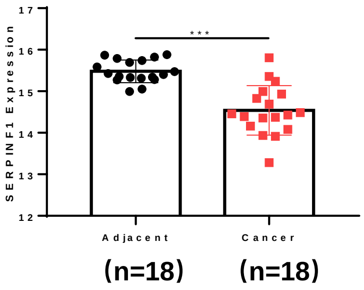

Supplementary Figure 2. PEDF mRNA level is downregulated in NPC tissues compared with adjacent tissues from GSE53819. The expression levels of PEDF mRNA in NPC adjacent tissues and cancer tissues from GSE53819.

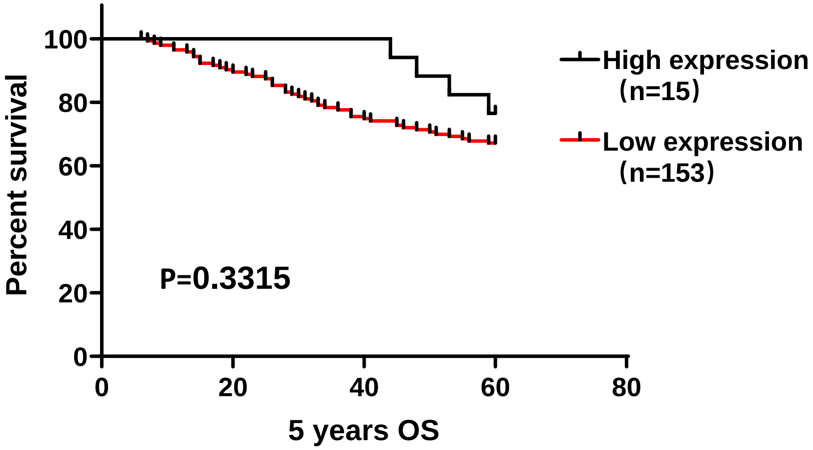

Supplementary Figure 3. No Significant correlation between PEDF and patient outcomes in NPC patients' 5-years survival analysis. Kaplan-Meier analysis of 5-years overall survival (OS) in a set of 168 nasopharyngeal cancer patients (n=168) according to PEDF expression.

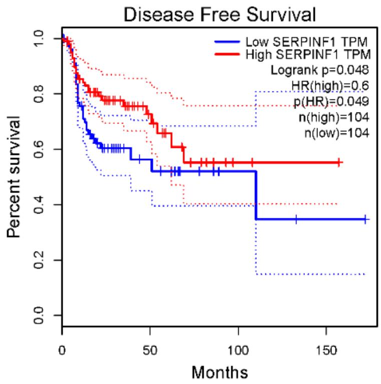

Supplementary Figure 4. High PEDF expression prolongs the overall survival in head and neck squamous cell carcinoma. GEPIA web tool was searched for the disease-free survival (P = 0.049, HR = 0.6) of HNSCC patients. HNSCC: head and neck squamous cell carcinoma

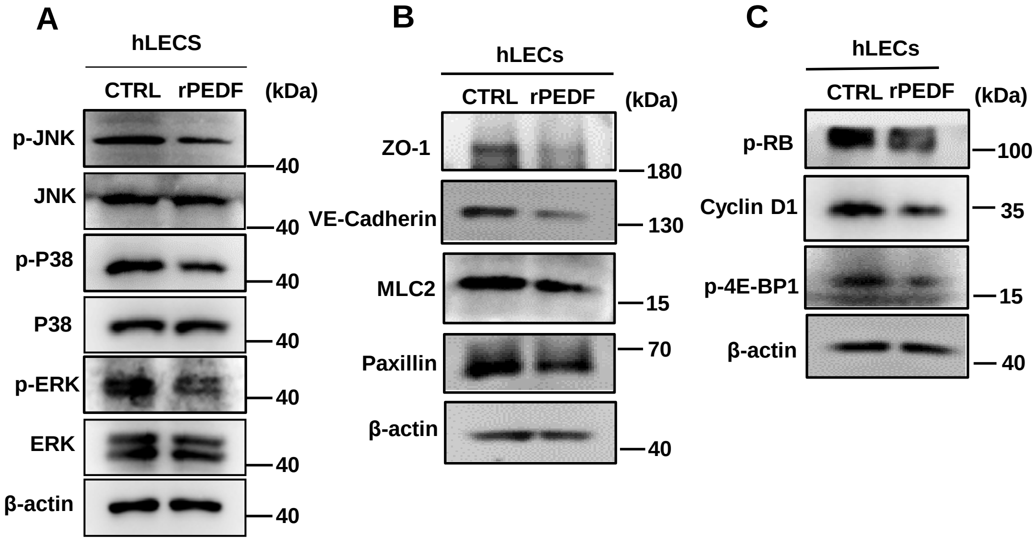

Supplementary Figure 5. PEDF inhibits MAPK signaling, migration, and proliferation markers in hLECs. The hLECs were treated with 640nM rPEDF for 48 hours, then (A) MAPK signaling protein (B) cell junction, migration, (C) proliferation markers were detected by western blot, β-actin was used as the loading control.

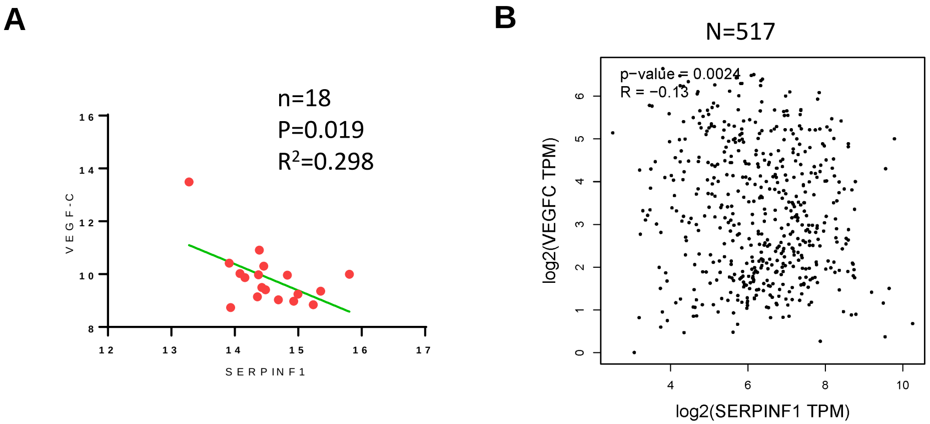

Supplementary Figure 6. PEDF is negatively correlated with VEGF-C mRNA in nasopharyngeal carcinoma and head and neck squamous cell carcinoma. (A) Correlation analysis of PEDF and VEGF-C mRNA expression level in NPC from GSE53819. (B) Correlation analysis of PEDF and VEGF-C mRNA expression level in HNSCC determined by the GEPIA web tool. HNSCC: head and neck squamous cell carcinoma

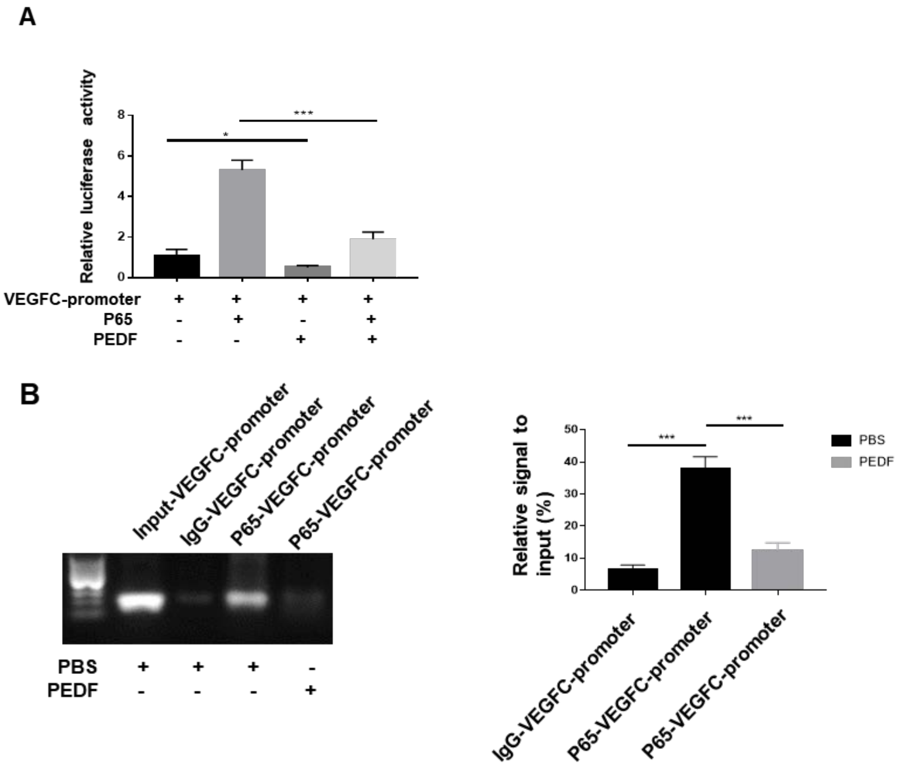

Supplementary Figure 7. The detachment of p65 from the promoter of VEGF upon PEDF overexpression. (A) Luciferase reporter assays of VEGFC promoter transcriptional activity. S18 cells were infected with P65-overexpressing plasmid or PEDF-overexpressing plasmid, VEGFC promoter-luciferase reporter plasmid, and Renilla luciferase plasmid for 36 h, followed by fluorescence detection. Renilla luciferase served as the transfection control. Bars represent the mean ± SD of three independent experiments; *** $p < 0.001$. (B) CHIP assays were performed to verify the detachment of P65 binding to the VEGFC promoter upon PEDF treatment. Lane 1: PCR product from input DNA; Lane 2: PCR product from immunoprecipitated by normal IgG; Lane 3: PCR product derived from immunoprecipitation by an anti-P65 antibody; Lane 4: After PEDF treatment in S18 for 36h, PCR product derived from immunoprecipitation by an anti-P65 antibody. Statistical analysis of ChIP assays (right).

Supplementary Table 1. Correlation of PEDF expression and clinicopathological characteristics of patients with NPC.

Characteristics	Number	Expression of PEDF		P-value
		Low	High	
**Age**				
≤ 48	92	83	9	0.669
> 48	76	70	6	
**Gender**				
Female	125	114	11	0.921
Male	43	39	4	
**Clinical stage**				
I+II	47	38	9	0.004
III+IV	121	115	6	
**T classification**				
T1-T2	76	65	11	0.022
T3-T4	92	88	4	
**N classification**				
N0	33	28	5	0.050
N1	51	44	7	
N2-N3	84	81	3	
**WHO histological**				
Type I	151	136	15	0.173
Type II-III	17	17	0	
**Loco-regional relapse**				
Yes	18	18	0	0.160
No	150	135	15	
**3 years survival**				
Yes	130	115	15	0.028
No	38	38	0	
**5 years survival**				
Yes	116	104	12	0.336
No	52	49	3	
