# Supplementary material for: PEDF inhibits lymphatic metastasis of nasopharyngeal carcinoma as a new lymphangiogenesis inhibitor
Source: Cell Death Dis. 2021 Mar 17;12(4):295. doi: 10.1038/s41419-021-03583-1 (PMC7969934; doi:10.1038/s41419-021-03583-1)
Supplement: Supplementary file 2 — Supplementary Table 1-editable [file 41419_2021_3583_MOESM2_ESM.docx]

Supplementary Table 1. Correlation of PEDF expression and

clinicopathological characteristics of patients with NPC.

| **Characteristics** | **Number** | **Expression of PEDF** | | **P-value** |
| --- | --- | --- | --- | --- |
|  |  | **Low** | **High** |  |
| **Age** |  |  |  |  |
| ≤ 48 | 92 | 83 | 9 | 0.669 |
| > 48 | 76 | 70 | 6 |  |
| **Gender** |  |  |  |  |
| Female | 125 | 114 | 11 | 0.921 |
| Male | 43 | 39 | 4 |  |
| **Clinical stage** |  |  |  |  |
| I+II | 47 | 38 | 9 | 0.004 |
| III+IV | 121 | 115 | 6 |  |
| **T classification** |  |  |  |  |
| T1-T2 | 76 | 65 | 11 | 0.022 |
| T3-T4 | 92 | 88 | 4 |  |
| **N classification** |  |  |  |  |
| N0  N1 | 33  51 | 28  44 | 5  7 | 0.050 |
| N2-N3 | 84 | 81 | 3 |  |
| **WHO histological** |  |  |  |  |
| Type I | 151 | 136 | 15 | 0.173 |
| Type II-III | 17 | 17 | 0 |  |
| **Loco-regional relapse** |  |  |  |  |
| Yes | 18 | 18 | 0 | 0.160 |
| No  **3 years survival**  Yes  No  **5 years survival**  Yes  No | 150    130  38    116  52 | 135    115  38    104  49 | 15    15  0    12  3 | 0.028      0.336 |
